# Silicon-Based Anode of Lithium Ion Battery Made of Nano Silicon Flakes Partially Encapsulated by Silicon Dioxide

**DOI:** 10.3390/nano10122467

**Published:** 2020-12-09

**Authors:** Yonhua Tzeng, Raycheng Chen, Jia-Lin He

**Affiliations:** Department of Electrical Engineering, National Cheng Kung University, One University Road, Tainan City 70101, Taiwan; q16061149@mail.ncku.edu.tw (R.C.); q16071194@mail.ncku.edu.tw (J.-L.H.)

**Keywords:** silicon, silicon oxide, CNT, anode, lithium ion battery

## Abstract

Ubiquitous mobile electronic devices and rapidly increasing electric vehicles demand a better lithium ion battery (LIB) with a more durable and higher specific charge storage capacity than traditional graphite-based ones. Silicon is among the most promising active media since it exhibits ten times of a specific capacity. However, alloying with lithium by silicon and dissociation of the silicon-lithium alloys induce high volume changes and result in pulverization. The loss of electrical contacts by silicon with the current collector of the anode causes rapid capacity decay. We report improved anode cycling performance made of silicon flakes partially encapsulated by silicon dioxide and coated with conductive nanocarbon films and CNTs. The silicon dioxide surface layer on a silicon flake improves the physical integrity for a silicon-based anode. The exposed silicon surface provides a fast transport of lithium ions and electrons. CNTs and nanocarbon films provide electrical connections between silicon flakes and the current collector. We report a novel way of manufacturing silicon flakes partially covered by silicon dioxide through breaking oxidized silicon flakes into smaller pieces. Additionally, we demonstrate an improved cycling life and capacity retention compared to pristine silicon flakes and silicon flakes fully encapsulated by silicon dioxide. Nanocarbon coatings provide conduction channels and further improve the anode performance.

## 1. Introduction

Lithium ion battery (LIB) is the most studied battery with the least technical barriers for emerging and high demanding energy storage needs for mobile devices. Long-range electric vehicles, long-lasting silent submarines [1,2,3], and renewable energy storage are examples. LIBs are powering modern electric vehicles (e.g., GM and Tesla electric cars and trucks) and electronic devices (e.g., mobile phones, notebook computers). However, to meet high challenging demands in the future, there is much room for improving the cycling durability and storage capacity per unit weight and volume of advanced LIBs. For example, it is desirable to upgrade hybrid electric vehicles (HEVs) to plug-in HEVs and pure-electric vehicles [4]. Solar energy generated during the sunny daytime and windmill energy generated at a windy time need to be stored and made available later when energy is needed. For these applications, LIBs must store more energy per unit volume and weight, and must repeat the charging and discharging operation for thousands of times.

The Sony Corporation introduced the first commercial Li-ion battery in 1991. It has a LiCoO_2_ cathode and a carbon anode [5]. When charges are stored, lithium ions move out of the cathode and are inserted inside the anode active medium, which is mostly graphitized carbon in traditional LIBs. Stored charges released by lithium ions exit from the anode and travel back to the cathode. In the external circuit, an electrical current, which is equal to the lithium ion current in the electrolyte, flows. In addition to the anode, many research groups are investigating cathodes, electrolytes, etc.

A good LIB anode must be stable and allow as much lithium as possible to repetitively insert and depart from the anode. It is required to maximize the cell energy density, power density, and cycle life [6]. For high-power applications, low-resistance and fast Li-ion transport within the anode is also necessary. The modern Li-ion battery industry uses mainly a graphite-based anode. In order to conserve a limited amount of lithium in a packaged battery cell, both the formation of robust solid-electrolyte-interphase (SEI) film and the minimization of the irreversible lithium compounds on the anode are desirable for high performance LIBs.

Lithium ions are “stored” in the carbon-based anode via intercalation in layered carbons such as graphite, adsorption on hard carbons, and binding with hydrogen atoms in hydrogenated carbons [4,7,8,9,10]. Mesocarbon-microbead (MCMB) has a high theoretical capacity of 372 mAh/g and low potential profile (0–0.3 V vs. Li/Li^+^). MCMB has been a standard anode material in modern commercial LIBs.

The specific capacity of 372 mAh/g for MCMB is not enough for high-demanding applications. Materials such as Sn, Sb, Si, and Ge [11,12,13,14] “stores” charges by reactions with lithium to form alloys. Among these, silicon has a much higher theoretical specific capacity of approximately 4200 mAh/g (ca. Li_4.4_Si) than MCMB and other anode materials [11,12,13]. However, silicon expands in volume by as much as 380% when it forms silicon-lithium alloys. The expansion-contraction of silicon causes irreversible mechanical damages to the silicon-based electrode. Pulverized silicon loses electrical contacts with the current collector. Electrons cannot transport from silicon to the current collector. Charge balance restricts positively charged lithium ions from departure from silicon and travelling to the cathode. Silicon thus loses the charging and discharging capability. In addition, new SEI grows on fresh silicon surfaces formed by the broken silicon. The SEI consumes additional Li. When Li in the battery is exhausted to a low level, the battery fails. How to retain electrical contacts among silicon particles with the current collector and to reduce the amount of irreversible SEI by reducing the fresh silicon surface in touch with the electrolyte are among the challenges. We wish to prevent the pulverization of silicon. If it is not possible, the pulverized silicon should be isolated from touching the electrolyte. However, the pulverized silicon must remain in electrical contact with the current collector.

Novel methods of minimizing the loss of electrical contacts of anode due to irreversible mechanical damages of the silicon-based anode have been invented, so that as much as possible electrical contacts are retained even if the active medium in the anode suffers from mechanical damages. We developed innovative processes to grow electrically conductive carbon films, carbon nanotubes, carbon fibers, and graphene nanowalls on the surface of silicon particles and flakes. These carbon nanostructures provide electrical conductivity between the neighboring silicon particles and flakes even after silicon breaks into multiple smaller pieces due to volume changes of silicon during repetitive charging and discharging. The charge storage capacity higher than 2000 mAh/g was reported after repetitive charging and discharging for more than 100 times [14,15,16]. The challenge in maintaining electrical contacts between silicon particles and between the anode and the current collector is feasible. The increased silicon surface area due to pulverization of silicon particles causes a significant challenge in the silicon-based anode. Electrons in the anode exit through the current collector to the external circuit rather than leaking into the electrolyte. Therefore, an interfacial layer known as SEI forms on the surface of active media in the anode, which is in touch with the electrolyte to allow lithium ions to penetrate it but not the electrons. The formation of SEI consumes the fixed supply of lithium in a closed system of a real-world LIB. The increased silicon surfaces may consume lithium to the extent that a LIB loses its charge storage capacity and fails prematurely. The growth of additional SEI on pulverized silicon is not fully reversible. Only a fraction of lithium used for forming SEI is recycled. The ratio of the released lithium to what enters the anode is referred to as the Coulombic efficiency (CE). At the beginning, a large area of SEI needs to form in a fresh anode. The Initial Coulombic efficiency (ICE) corresponding to the first discharge-charge cycle is usually the lowest among all cycles. The consumption of lithium accumulates and the fixed amount of lithium in the LIB gradually becomes exhausted.

When a surface silicon atom reacts with lithium from the electrolyte to form alloys, the volume of the silicon-lithium alloys would expand outwards from the surface freely with little adverse effects to the integrity of the silicon. When lithium diffuses into silicon to react with a bulk silicon atom and form an alloy, atoms surrounding this alloy must yield to the volume expansion. This creates stress leading to possible pulverization. Silicon particles of sub-100 nm, especially those of the size of low tens of nanometers are less likely to pulverize since the ratio of the surface atoms to bulk atoms is large. However, the manufacturing cost for silicon nanoparticles increases with decreasing particle sizes. Sub-100 nm silicon particles are more favorable than the micron-sized silicon as an anode material for LIB. In the real world, battery production needs many tons of anode materials. Unless an economic means of producing sub-100 nm to deep-sub-100 nm sized silicon nanoparticles is available, the costs of silicon nanoparticles remain a negative factor to consider against competition with other anode materials.

In order to accommodate the expanding silicon, extra space around individual silicon particles is preserved. For example, the silicon core in the hollow non-silicon shell exhibits a long cycling life. Porous silicon exhibits similar benefits as core-shell silicon structures. Researchers used both top-down etching and bottom-up growth processes to fabricate nanoscale silicon wires and rods for LIB anode applications [17,18,19].

Other potential anode materials for LIBs based on alloying/de-alloying and conversion reactions include, for example, P (2596 mAh/g), Sn (960 mAh/g), Sb (660 mA h/g), Ge (1600 mAh/g), and transition metal oxide (TMOs). These are promising but not commercialized yet. Pure silicon anode is not ready for commercial use yet due to mainly the issue of silicon volume expansion. Instead, LIB manufacturers add 5–10% silicon to the traditional graphite based anode such as MCMB for commercial uses.

In addition to the pure silicon-based anode for LIB, researchers around the world are investigating silicon oxide, silicon carbide, and silicon nitride for anode. Compared to these materials, pure silicon is the weakest in terms of physical strength against pulverization. On the other hand, the higher the fraction of oxygen is in the silicon oxide, the harder the silicon oxide. Unfortunately, the higher the oxygen fraction, the lower the charge storage capacity the silicon oxide exhibits. A high charge storage capacity and good physical integrity are required simultaneously. A mixture of graphite with partially oxidized silicon, i.e., SiO*_x_*, with x being less than 2 is used in commercial LIBs. SiO*_x_* also suffers from pulverization after long cycling. It also exhibits a low initial Coulombic efficiency than pure silicon due to the tendency for silicon oxide to form irreversible Li-salt. SiO*_x_* also suffers from a lower capacity compared to pure silicon and higher electrical resistance for conducting charging and discharging currents. Nevertheless, it provides good physical integrity and is among the best performing anode materials for LIB from the viewpoint of durable cycling performance.

The research and development of better and economic SiO*_x_* has attracted many researchers around the world. In practice, oxidized silicon anode materials being studied include (i) SiO-based anode materials, (ii) SiO_2_-based anode materials, (iii) non-stoichiometric SiO*_x_*-based anode materials, and (iv) Si–O–C based anode materials. Liu et al. published a comprehensive review of silicon oxides as a promising family of anode materials for lithium ion batteries. Atomic models for amorphous Si, interfacial silicon sub-oxide, SiO_2_, and SiO are presented in [20,21].

SiO is thermodynamically unstable and has the tendency of disproportionating into Si and SiO_2_. Nanoscale silicon is included in a framework of SiO_2_. The total capacity of Si and SiO_2_ is less than that of pure Si but the volume expansion of a mixture of Si and SiO_2_ during cycling is not as severe as that for pure Si. The compromise between a lower capacity and the less volume changes makes SiO*_x_* attractive for long-life LIB applications. Yang et al. studied the performance of SiO, SiO_0.8_, and SiO_1.1_ and found that the capacity decreases with increasing oxygen contents but with improving cycling performance. SiO_0.8_ had a reversible capacity of about 1600 mAh/g [22]. This result further inspired world-wide interest of scientists in studying SiO*_x_*-based anode materials.

SiO_2_ has a high theoretical specific capacity of 1965 mAh/g. However, SiO_2_ has a high electrical resistivity and low diffusivity for Li^+^. Porous silicon oxide and nanoscale SiO_2_ overcome this problem. Jiao et al. synthesized a 400 nm SiO_2_ ball by the sol-gel method. The initial specific capacity was 622 mAh/g with a low ICE being 54.8%. After discharge-charges for 500 cycles, the specific capacity became 877 mAh/g. Liang et al. ball milled SiO_2_ down to smaller than 1 µm in size and reported a 600 mAh/g specific capacity after 150 cycles of discharges and charges [23,24].

The LIB anode containing SiO*_x_*, *x* < 2, is nowadays a commercial product. The high cost for manufacturing SiO*_x_* makes new materials and nanostructures of the same functions desirable. Silicon flake is an inexpensive by-product of silicon crystal and wafer manufacturing processes. The typical dimensions of the as-received silicon flake we use is about 100 nm in thickness and 800–1000 nm in width and length.

By thermally oxidizing a 10 nm surface layer of a silicon flake to form silicon dioxide, the remaining silicon of about 80 nm in thickness and 800–1000 nm in width and length is fully encapsulated by silicon dioxide. Only about 20% by volume of the as-received silicon flake is oxidized to form silicon dioxide. This is an inexpensive process to fabricate silicon-silicon-dioxide nanostructures compared to melting silicon under controlled oxidation environments to form SiO*_x_*.

However, silicon dioxide exhibits a very low lithium diffusion coefficient and a very high electrical resistance for electron transport. Some theoretical calculation indicates that dopings and defect engineering might improve the lithium diffusion in oxide materials [25,26]. If this technique is successful in economic mass production, silicon dioxide can serve as a good mechanical support for better physical integrity of the silicon-based anode, while at the same time provide a reasonable transportation of lithium ions and electrons.

Alternatively, we can break a 100 nm thick silicon flake of, for example, 1000 by 1000 nm in size, into two smaller silicon flakes of 500 by 1000 nm or more silicon flakes of even smaller sizes. The fresh silicon surface is exposed on the cross-sectional plane along the broken line. The transportation of electrons and lithium ions to and from the silicon active medium becomes much easier.

The remaining silicon dioxide remains to be an electrical insulator and the fresh silicon surface is not in direct contact with the other silicon surface and the current collector in the anode. Electrically conductive nanocarbon coatings on both the silicon dioxide and the fresh silicon surface serve to improve the electrical connections between the exposed fresh silicon surface on neighboring broken oxidized silicon flakes and the current collector of the anode.

Carbon is a very stable anode material for LIBs. It is natural that the carbon-based nanostructure is considered for application to the anode of LIBs [27]. Therefore, silicon-oxide-carbon composites are considered promising for resolving the challenging issues related to insufficient internal electrical conductivity of the silicon-oxide-based anode. Many means of applying nanocarbons for improving electrical connectivity of the silicon-based anode also apply to the silicon-oxide-based anode. For example, graphene and graphene oxide wrap around silicon nanoparticles [28,29]. Filling voids in the anode by CNTs provides additional electrical connections [30,31]. Pyrolysis of polymeric coatings on silicon nanoparticles forms solid or porous carbon interfacial layers [32,33]. Graphene nanowalls were grown directly and vertically on the silicon surface as extra electrical interconnections [16]. Nanocarbon films and CNTs deposit by means of thermal CVD, as well [34,35]. An ideal carbon coating on silicon or silicon oxide nanoparticle should provide excellent electrical conductivity, the necessary buffer space for the volume expansion of active media, and isolate the fresh surface of pulverized silicon and silicon oxide from reactions with the electrolyte to form additional SEI. The carbon coating must be suitable for mass-production. Many techniques for synthesizing CNTs have also been reported [36,37,38].

In this paper, we report our investigation into Si flakes partially covered by SiO_2_ as an active medium for LIB anode. The silicon flakes we use are about 100 nm thick and about 800–1000 nm in width and length. The SiO_2_ is about 10 nm in thickness. Although, the specific capacity of SiO_2_ is much less than that of pure Si, the physical strength of SiO_2_ is stronger than Si. The thermally oxidized silicon encapsulates the silicon flakes and improves the physical strength and integrity of silicon during charge-discharge cycling.

## 2. Materials and Methods

Silicon flakes of about 100 nm thick and 800–1000 nm in length and width were provided by one of our industrial collaborators at AUO Crystal, Corp., which is a silicon wafer manufacturing company in Taichung, Taiwan. The silicon flakes are part of silicon containing waste slurry generated by cutting silicon ingots and from other silicon wafer manufacturing processes. The company uses an economic and proprietary chemical process to recover and purify the silicon flakes from the slurry.

The morphology and the structure of the nanocarbon coated silicon were observed by scanning electron microscopy (SEM, Hitachi-SU8000, Taipei, Taiwan). Scanning transmission electron microscopy (STEM, JEOL JEM-2100F Cs, Taipei, Taiwan) was used to reveal the silicon dioxide layer on a silicon flake. The acceleration voltage is 200 kV.

A Horiba Scientific Raman (Hsinchu, Taiwan) system with a green laser at 532 nm and laser power at 450 mW was used to measure Raman spectra. The laser beam was focused on the sensor surface in an area of about 10 µm in size. Raman spectra reveals the nanostructures of nanocarbons.

Coin cells (CR2032) were fabricated for testing the performance of the anode. Nanocarbon coated silicon flakes after having been broken into smaller pieces by ball milling were mixed with carbon black and sodium carboxymethyl cellulose (NaCMC) in DI water with a weight ratio of 6:3:1. The slurry was stirred homogenously and then applied on a 10 µm thick copper foil by a doctor blade. The thickness of the active materials was 30 µm. After the electrode was dried at 60 °C for 12 h, the electrode was cut into small pieces with a diameter of 12 mm. Then, the electrode was put into an Ar-filled glove box with residual oxygen and moisture contents of less than 0.5 ppm to assemble the coin cell. The electrolyte was the 1 M LiPF6 in ethylene carbonate (EC) and dimethyl carbonate (DMC) (1:1 *v*/*v*) solution. The charge-discharge cycling performance was analyzed by a battery testing system (BAT-750B).

**Figure 1:**
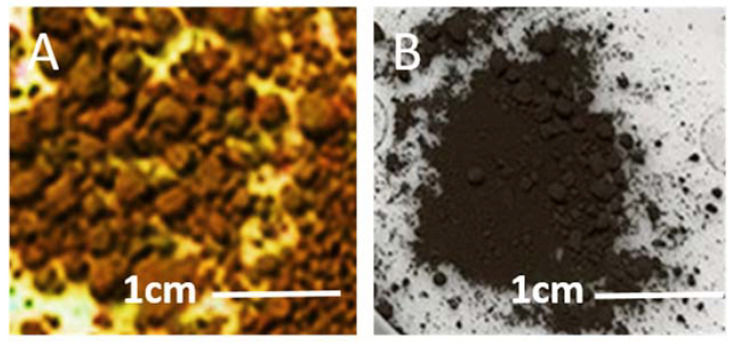
Optical micrographs of (**A**) as-received silicon flakes in brownish color and (**B**) wet oxidized silicon flakes in dark grey color.

Figure 1A shows pristine silicon flakes in brownish color. Figure 1B shows oxidized silicon flakes in dark grey color. The oxidization temperature was 850 °C. Water vapor was bubbled into the furnace by argon gas through a water set at 25 °C. Table 1 shows the parameters for the wet oxidation process.

**Figure 2:**
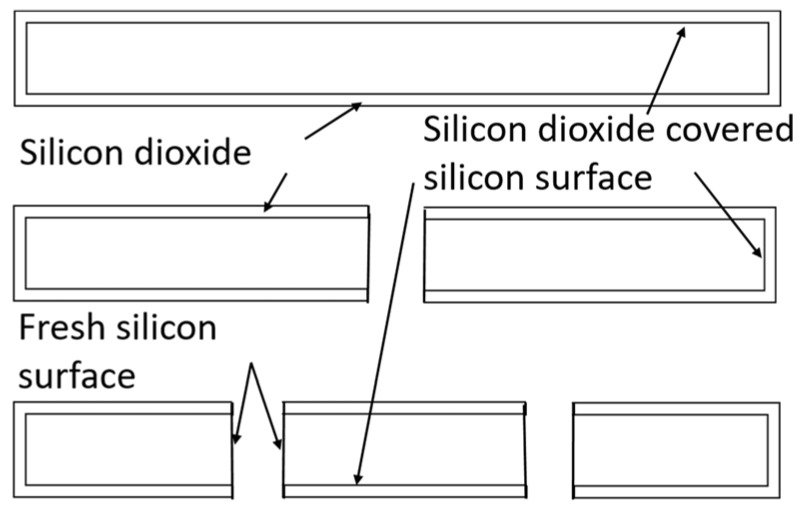
Cross-sectional view of an oxidized silicon flake and smaller pieces of a broken oxidized silicon flake with fresh silicon surface not covered by silicon dioxide.

Figure 2 shows the schematic diagrams illustrating a pristine oxidized silicon flake and oxidized silicon flakes having been broken into smaller silicon flakes. We used the ball milling technique to break individual oxidized silicon flakes into two or more smaller flakes. The thin and flat geometry of the silicon flakes results in a high probability for silicon flakes to break into two or more pieces of smaller silicon flakes of the same thickness as the original silicon flake of about 100 nm thick but smaller in width and length than the original flake of 800 nm in length and width. When a flake breaks into two pieces, only the silicon surface on the broken plane is exposed. Oxidized silicon covers the remaining silicon surface. The exposed silicon surfaces allow lithium to react with silicon and form lithium-silicon alloys. Additional lithium can diffuse deeper into the silicon flakes for further alloying reactions. Lithium can diffuse through the thin SiO_2_ layer but at a much lower diffusion coefficient than that for silicon. The SiO_2_ layer provides additional physical strength to retain the silicon integrity even if the silicon inside the SiO_2_ layer breaks into pieces or pulverizes. For the silicon volume to expand, the overall thickness of flat silicon including thin SiO_2_ on both sides may increase. Silicon may also expand in volume and extend outside of the SiO_2_ encapsulation from the window along the breaking plane. An oxidized silicon flake may be broken into three or more pieces. More of the silicon surface reacts with lithium to form alloys.

**Figure 3:**
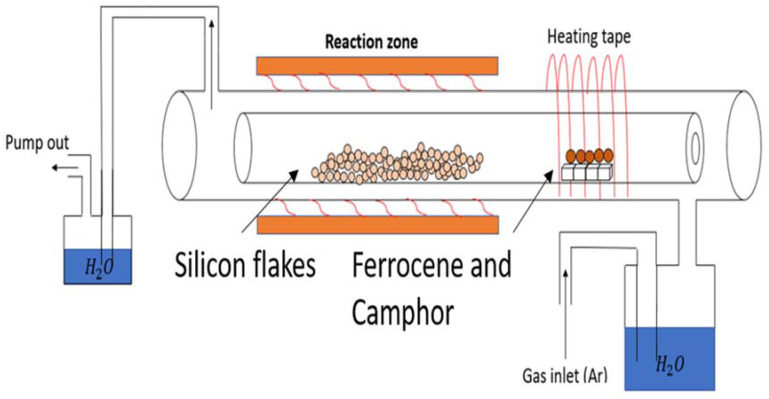
Schematic diagram of a thermal Chemical Vapor Deposition (CVD) system for the deposition of nanocarbons on silicon flakes partially covered by silicon dioxide.

Figure 3 shows a thermal CVD reactor used for the coating of CNTs and nanoscale carbons. After oxidized silicon flakes were broken by ball milling into smaller pieces to expose the fresh silicon surface, we deposited CNTs and nanoscale carbons by thermal CVD using ferrocene and camphor as precursors. Ferrocene contains iron, which served as both an effective catalyst and a carbon source for the synthesis of CNT [39,40,41,42]. Camphor is an additional carbon source [43]. The precursors were vaporized by heating to 16 °C. Argon flew through a water bubbler, which was set at room temperature and carried water vapor through the precursor heating zone and then to the CVD reaction chamber where the temperature was set at 700 °C. Electronic temperature controllers were used to control both heating zones. Broken oxidized silicon flakes after ball milling were placed in the heated CVD zone. The container for silicon flakes partially covered by silicon dioxide was rotated in order to achieve uniform coatings. A mechanical roughing pump evacuated the chamber to about 50 mTorr. Argon gas was used to fill the reaction chamber. After purging, the roughing pump was switched off with the exhaust gas going through the second water bubbler. The thermal CVD process was carried out at one atmospheric pressure. After the heating zone reaches 700 °C, the precursors were heated to 160 °C. The Ar flow rate was typically set at 400 sccm. An example of the precursor loading included 0.7 g silicon flakes, 4 g camphor, and 1.8 g ferrocene. After the process, all precursors vaporized. Iron, which was exposed to water vapor was oxidized. The growth time was 15 min. The nanocarbon deposition was about 10% by weight of the coated silicon flakes with the surface partially covered by silicon dioxide.

The low temperature thermal CVD in water vapor environments using ferrocene and camphor as precursors deposits multiple phases of nanocarbons including CNTs, carbon fibers, and graphitic carbon films. There is no exposed metallic iron in the oxidized silicon flakes with CNTs and nanocarbon deposition. Table 2 summarizes the CVD parameters.

## 3. Results and Discussion

**Figure 4:**
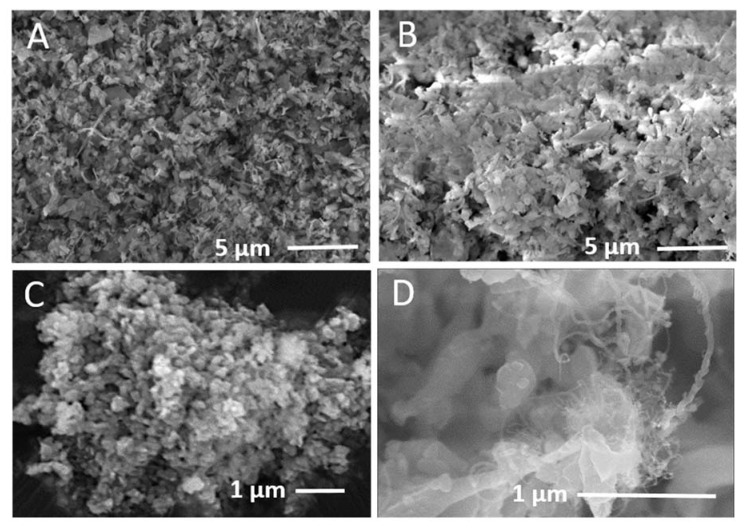
SEM images of (**A**) pristine silicon flakes; (**B**) oxidized silicon flakes; (**C**) oxidized silicon flakes after having been broken into smaller pieces by ball milling; and (**D**) nanocarbon deposition on oxidized silicon flakes which have been broken into smaller pieces by ball milling.

Figure 4 shows SEM images of before and after processes of silicon flakes. Figure 4A is an SEM image of pristine silicon flakes. Figure 4B is an SEM image of the oxidized silicon flakes. Silicon dioxide is an electrical insulator. The secondary electron yield from silicon dioxide under SEM observation is higher than that from the semiconducting silicon. The brighter image of Figure 4B than that of Figure 4A is due to the surface of pristine silicon flake having been oxidized and covered by a layer of electrically insulating silicon dioxide. Figure 4C is an SEM image of the oxidized silicon flakes having been broken into smaller pieces by ball milling. The 100 nm thick silicon flakes before ball milling are about 800–1000 nm in width and length. Figure 4C shows that ball milling breaks individual silicon flakes into flakes of 200–500 nm in width and length. For a silicon flake with surface silicon dioxide, electrons cannot transport from the silicon surrounded by silicon dioxide to the current collector of the anode of a LIB. The fresh silicon surface not covered by silicon dioxide was exposed when a silicon flake with its surface covered by silicon dioxide was broken into small pieces. Figure 4D shows an SEM image of ball milled smaller pieces of oxidized silicon flakes, which have been deposited with electrically conductive nanocarbons including CNTs. The nanocarbon content was about 10% by weight based on the measured weight of the oxidized silicon flakes before and after the nanocarbon deposition.

**Figure 5:**
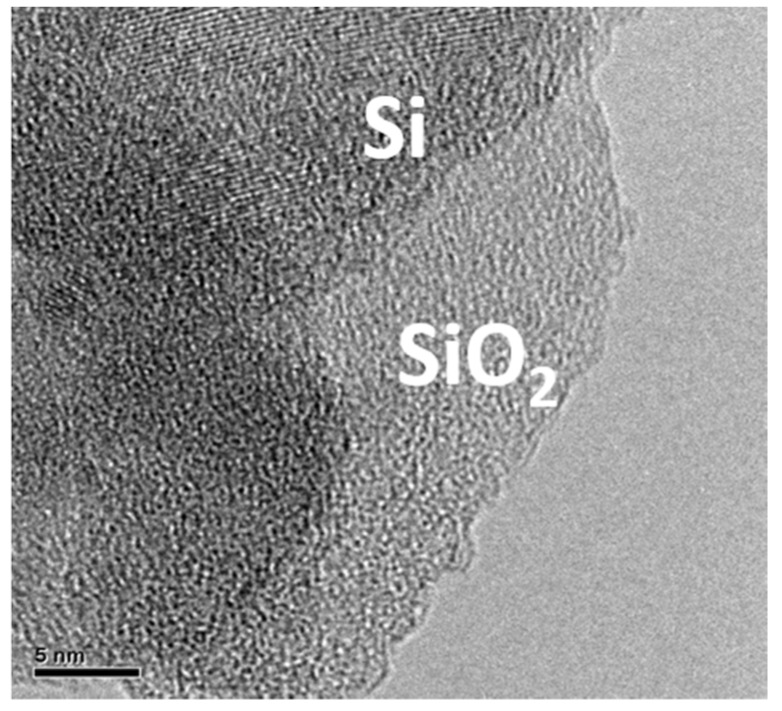
TEM image of an oxidized silicon flake after having been broken into smaller pieces by ball milling.

Figure 5 shows TEM images of an oxidized silicon flake after having been ball milled to break them into smaller flakes with an exposed fresh silicon surface. Figure 5 shows both crystalline silicon and amorphous silicon dioxide. The amorphous silicon dioxide indicated in Figure 5 is about 10 nm thick. This is consistent with the wet oxidation in low-pressure water vapor and at a low temperature. The thickness of the silicon dioxide increases with the oxidation time and temperature. With 10 nm silicon dioxide on each side of a silicon flake of 100 nm in thickness, the remaining silicon surrounded by silicon dioxide is about 80 nm in thickness. The optimal thickness of silicon dioxide suffices to enhance the physical integrity of the surrounded silicon and the anode it makes. Since oxidation increases the weight of a pristine silicon flake, it is not desirable to convert too much of the silicon into the silicon dioxide. The water in the bubbler is set at room temperature so that the oxidation proceeds at a reasonable low rate and the oxide thickness is determined by adjusting the oxidation time. At room temperature, the water vapor pressure is much lower than the argon carrier gas pressure at one atmosphere in the oxidation chamber.

Electrically conductive graphitic nanocarbon coatings were deposited by thermal CVD using argon as a carrier gas, which passed through a water bubbler at room temperature to carry water vapor to the reaction chamber. Ferrocene and camphor were used as the precursors. Ferrocene contains iron, which serves as a catalyst for CNT growth. Ferrocene also serves as a carbon source for CNT and nanocarbon growth. Camphor provides additional carbon for CNT growth. The vapor of ferrocene and camphor, which were heated to 160 °C was carried by the argon carrier gas to the CVD reaction zone set at 700 °C. The reaction time was adjusted to achieve a desired weight percentage of nanocarbons being deposited on oxidized silicon flakes with partially exposed fresh silicon surface. The nanocarbon coating used for this experiment was about 10% of the total weight.

**Figure 6:**
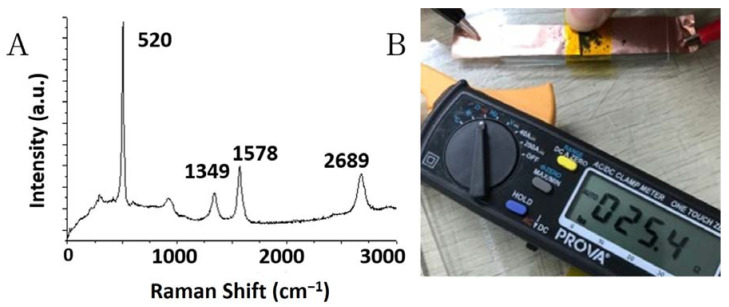
(**A**) Raman spectrum of nanocarbon coating and (**B**) resistance measurement of nanocarbon coated silicon flakes.

Figure 6A shows a Raman spectrum of nanocarbons deposited on oxidized silicon flakes after having been broken into smaller pieces by ball milling. Raman excitation was by green laser at 532 nm. The Raman spectrum displays three characteristic Raman peaks at 1349, 1578, and 2689 cm^−1^ corresponding to the D-band, G-band, and 2-D band, respectively. These Raman peaks are characteristic of graphitic nanocarbons such as CNT, carbon nanofibers, and electrically conductive nanocarbon films. Raman scattering data, the SEM images shown in Figure 4D, the weight increase after thermal CVD of nanocarbons, and the electrical conductivity measurement, jointly show that electrically conductive nanocarbons have been deposited on oxidized silicon flakes with the exposed fresh silicon surface. The 10% by weight coating of nanocarbons provided low internal resistance needed for the anode to perform. Resistance of the nanocarbon coating was measured by placing a fixed amount of silicon flakes on a silicon dioxide plate with two copper electrodes on top of the powder near both ends. The measured total resistance between two copper electrodes was on the order of tens of Ohms, which was several orders of magnitude lower than the resistance measured from the pristine silicon flakes by the same method. The pristine silicon flakes were measured to exhibit multiple millions Ohms, which was usually out-of-range of the resistance meter.

**Figure 7:**
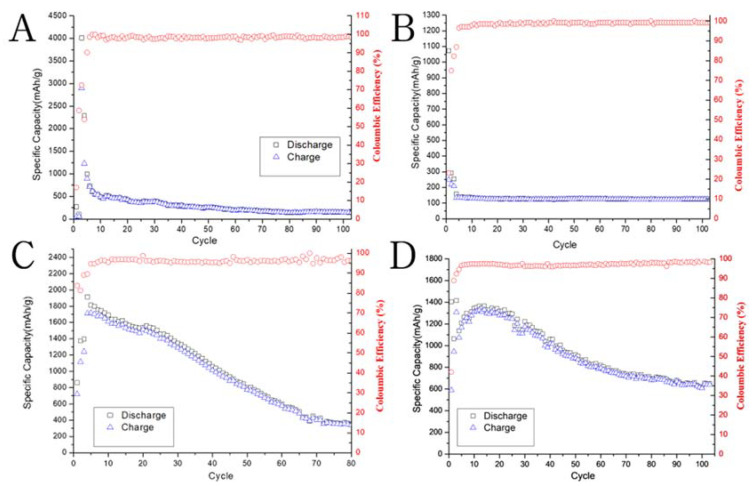
Cycling performance of lithium ion battery (LIB) half-cells with the anode made of (**A**) pristine silicon flakes; (**B**) oxidized silicon flakes; (**C**) broken pieces of oxidized silicon flakes; and (**D**) nanocarbon coated broken pieces of oxidized silicon flakes.

We assembled LIB half-cells for testing, using the broken oxidized silicon flakes as the anode material, following the same procedure presented in our prior publication [16]. We evaluated the cycling performance at the 0.02 C for the first three cycles and then 0.1 C for the remaining cycles. Figure 7A shows the rapid decay of specific capacity of the pristine silicon flake-based anode. Figure 7B shows that the specific capacity of oxidized silicon flakes is even lower beginning from the first cycle. The resistance of silicon dioxide is high and lithium diffusion through silicon dioxide is slow. Therefore, the poor cycling performance of the oxidized silicon flake-based anode is expected. Figure 7C shows a much improved specific capacity after the thermally oxidized silicon flakes are broken into smaller flakes with the fresh silicon surface exposed to the electrolyte for alloying with lithium. This demonstrates the success of the main novelty of this work, i.e., creating sub-100-nm thick silicon flakes with surface partially covered by silicon dioxide and its application to the LIB anode. Figure 7D shows how the deposition of CNT and nanocarbons further improves the specific capacity. After 100 cycles, the specific capacity retains about 600 mAh/g. By comparing the cycling performance displayed in Figure 7, the benefits of high specific capacity and long cycling life of oxidized flakes after ball milling to expose the fresh silicon surface and the subsequent deposition of conductive nanocarbons were clearly demonstrated.

## 4. Conclusions

The silicon-based anode for LIBs making use of silicon flakes that recovered from a semiconductor industrial waste has shown promising performance. The processes of wet oxidation and ball milling are inexpensive. A novel process of fabricating sub-100-nm thick silicon flakes, which are partially enclosed by the thermal silicon dioxide, while exposing the silicon surface for electron transport and alloying with lithium is reported. The thermal CVD of conductive nanocarbons improves the anode performance further. Optimization of the novel fabrication process for the silicon-oxide-based LIB anode is currently undertaken. We hope that this report may inspire additional novel ideas and collaboration towards producing economic and high performance active silicon-based media for the LIB anode.

## Figures and Tables

**Table 1:** Parameters for the wet oxidation process of silicon flakes.

Wet Oxidation	Ar (sccom)	Temperature (°C)	Time (min)
Step 1	400	25	20
Step 2	400	25–700	20
Step 3	400	700–850	10
Step 4	400	850	240

**Table 2:** Nanocarbon thermal CVD process parameters.

Nanocarbon Thermal CVD	Ar (sccm)	Furnace Temperature (°C)	Time (min)	Heating Tape Temperature (°C)
Step 1	400	25	30	25
Step 2	400	25–>700	10	25
Step 3	400	700	3.5	160
